# The Clinical Utility of No-Touch Saphenous Vein Grafting as a Second Conduit in Multivessel Coronary Artery Bypass Surgery

**DOI:** 10.5761/atcs.oa.25-00151

**Published:** 2025-11-14

**Authors:** Hiroshi Kurazumi, Ryo Suzuki, Takato Nakashima, Ryosuke Nawata, Toshiki Yokoyama, Kazumasa Matsunaga, Bungo Shirasawa, Akihito Mikamo, Kimikazu Hamano

**Affiliations:** Division of Cardiac Surgery, Department of Surgery and Clinical Science, Yamaguchi University Graduate School of Medicine, Ube, Yamaguchi, Japan

**Keywords:** no-touch saphenous vein graft, coronary artery bypass, graft patency, internal thoracic artery, gastroepiploic artery

## Abstract

**Purpose:**

We aimed to compare the midterm outcomes of the no-touch saphenous vein graft (NT-SVG) as a second conduit with those of other graft types.

**Methods:**

We retrospectively reviewed 549 consecutive patients who underwent multivessel isolated coronary artery bypass grafting (CABG) with ≥2 distal anastomoses between 2002 and 2024. Five conduit groups for non-LAD grafting were analyzed: in situ internal thoracic artery (ITA), free ITA, conventional saphenous vein graft (cSVG), NT-SVG, and right gastroepiploic artery (rGEA). We analyzed conduit-specific patency and propensity score–matched patency between cSVG and NT-SVG.

**Results:**

The mean age was 68.6 ± 9.5 years, and 74% were men. Off-pump CABG was performed in 60.5% of cases, with a mean of 3.3 ± 0.9 distal anastomoses. Hospital mortality was 1.5%. Notably, the 5- and 10-year survival rates were 85.9% and 74.1%, respectively. Among 794 non-LAD grafts, the NT-SVG demonstrated a 5-year patency of 96.4%, which was significantly higher than that of cSVG (89.5%, p = 0.05) and rGEA (87.2%, p = 0.04), and equivalent to in situ ITA (94.4%) and free ITA (95.0%). The propensity score–matched analysis further demonstrated superior graft patency with the NT-SVG.

**Conclusions:**

The NT-SVG achieves a 5-year patency comparable to that of ITA grafts and superior to that of cSVG and rGEA, suggesting its potential as a promising option for non-LAD revascularization, pending further validation.

## Introduction

Coronary artery bypass grafting (CABG) remains the standard of care for multivessel coronary artery disease, offering superior survival and symptomatic relief compared with percutaneous coronary intervention in appropriately selected patients.^1–3)^ The dominance of the in situ internal thoracic artery (ITA) compared to that of the left anterior descending artery (LAD) is firmly established^4,5)^; however, the appropriate second conduit for non-LAD territories is still debated. Conventional saphenous vein grafts (cSVGs) are easily harvested but offer 10-year patency rates of 50%–60%, largely due to endothelial injury during preparation.^6)^ The no-touch (NT) harvesting technique preserves perivascular tissue, prevents overdistension, and minimizes endothelial trauma, offering better long-term outcomes.^7)^

In CABG procedures performed in Japan, the SVG is the second most commonly used conduit following the ITA.^8)^ The no-touch SVG (NT-SVG) has been explored earlier as a preferred second conduit,^9,10)^ and the 2018 European Society of Cardiology/European Association for Cardio-Thoracic Surgery guidelines on myocardial revascularization provide a Class IIa recommendation for NT-SVG harvesting as a means to improve graft outcomes.^11)^

Our institution introduced the NT-SVG technique in 2016. In this study, we aimed to compare the mid-term outcomes of NT-SVG with those of other graft types when used as a second conduit.

## Materials and Methods

### Study design and patient population

**Fig. 1:**
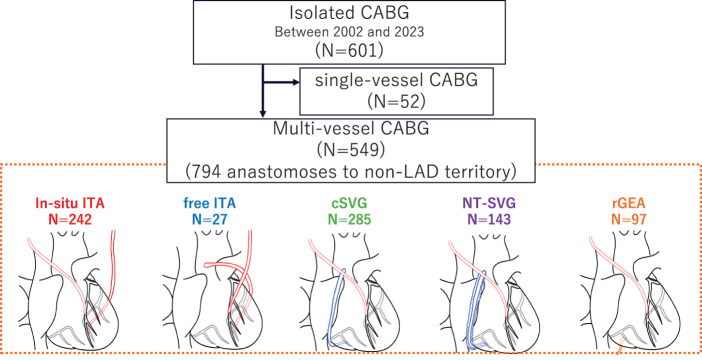
Study enrollment and group allocation. CABG: coronary artery bypass grafting; LAD: left anterior descending artery; ITA: internal thoracic artery; cSVG: conventional saphenous vein grafts; NT-SVG: no-touch saphenous vein grafts; rGEA: right gastroepiploic artery

In this single-center retrospective cohort study, we reviewed 601 consecutive, isolated CABG procedures undertaken between January 2002 and March 2023 at Yamaguchi University Hospital. The study population comprised all adult patients who underwent isolated CABG at our institution. Of these, 549 multivessel cases with ≥2 distal anastomoses were analyzed (**Fig. 1**).

### Surgical strategy for CABG

All operations were performed through a median sternotomy. Cardiopulmonary bypass was employed at the surgeon’s discretion in patients with poor ventricular function, hemodynamic instability during off-pump manipulation, or deeply located target vessels. The LAD was grafted with either left or right in situ ITA whenever feasible. For the circumflex territory, a second ITA was preferred; for the right coronary territory, the right gastroepiploic artery (rGEA) was used in young patients with ≥90% stenosis; otherwise, the SVG was selected. Since 2016, all SVGs have been harvested using the NT technique.

### Details of harvesting for CABG conduits

#### ITA harvesting

The left ITA was harvested in a skeletonized fashion using an ultrasonic energy device (HARMONIC SYNERGY; Ethicon, Somerville, NJ, USA). All side branches of the ITA were meticulously controlled: smaller branches were divided using simultaneous ultrasonic coagulation, whereas larger branches were secured with surgical clips before division to ensure complete hemostasis.

#### Conventional SVG harvesting

The great saphenous vein was harvested from the lower leg using an open conventional technique with skeletonization of the vein (removal of perivascular tissue). All venous side branches were carefully ligated to prevent bleeding along the conduit. The excised vein was gently flushed and manually distended with heparinized saline using a syringe to check for any branch leakage. During this manual distension, care was taken to keep the intraluminal pressure below 300 mmHg to avoid endothelial injury and conduit damage.

#### NT-SVG harvesting

The great saphenous vein was harvested from the lower leg. SVG was excised with 5 mm of surrounding perivascular adipose tissue (PVAT) left intact. All visible side branches were ligated. If none of the branches were visible, the surrounding fat was separated using an ultrasonic energy device (HARMONIC FOCUS+Scissors Handle; Ethicon). The harvested vein was then connected to a 4-Fr sheath inserted into the femoral artery and gently dilated for 10 min at systemic pressure.

### Wound closure methods at the graft harvest site

Among the 281 patients who underwent SVG harvesting using the conventional technique, the first 176 had the harvest site closed primarily without drain placement, whereas in the remaining 105, a 10-Fr silicone drain (J-VAC drain; Ethicon) was placed in the subcutaneous space before wound closure.

Among the 116 patients who underwent SVG harvesting using the no-touch technique, the first 105 had the harvest site closed with placement of a 10-Fr silicone drain, whereas in the remaining 11, the wound was closed primarily without drain placement, and a negative-pressure wound therapy system (Prevena; KCI, an Acelity company, San Antonio, TX, USA) was applied to the surface of the harvest site.

### Intraoperative graft flow measurement

Intraoperative graft flow was measured using a transit-time flowmeter. The principle of transit-time flowmeters has been described in previous studies.^12,13)^ Measurements were performed after protamine reversal at a systolic pressure of >100 mmHg and a heart rate <100 bpm. A 3-mm probe was used for all conduits, and mean graft flow (mGF) and pulsatility index (PI) were recorded.

### Data collection and endpoints

Overall survival and major adverse cardiac and cerebrovascular events (MACCE) were ascertained from clinical records and telephone interviews. The mean observation period was 6.5 ± 5.4 years. Graft patency was assessed by coronary angiography or cardiac computed tomography (CT) within one month postoperatively in patients without renal dysfunction. Graft occlusion was detected using coronary angiography or multislice CT angiography. According to the FitzGibbon criteria,^14)^ graft occlusion was considered when a conduit did not fill with contrast at all or when a string sign was found in any segment. Subsequent follow-up comprised annual outpatient visits, during which routine assessments—such as electrocardiography, chest radiography, and transthoracic echocardiography—were performed. If patients developed new symptoms or any abnormality was detected during these examinations, graft patency was further evaluated by coronary angiography or cardiac CT.

The primary endpoints were overall survival and MACCE in all 549 patients. The secondary endpoints were conduit-specific patency, mGF, and PI.

### Statistical analysis

Continuous variables are expressed as mean ± standard deviation. Survival and patency were analyzed using the Kaplan–Meier method (JMP Pro 16.1.0; SAS Institute Inc., Cary, NC, USA). A 2-sided p <0.05 was considered statistically significant.

## Results

### Patient characteristics

Study enrollment and group allocation are shown in **Fig. 1**. Notably, 549 patients underwent isolated multivessel CABG with at least two distal anastomoses at our institution between 2002 and 2023. Patient characteristics are shown in **Table 1**. The mean age of the cohort was 68.6 years, and approximately 74% of the patients were men. Traditional cardiovascular risk factors were highly prevalent: 51% had diabetes mellitus and 64% had dyslipidemia. Renal dysfunction was present in a minority of cases, with about 6% of patients receiving preoperative hemodialysis therapy. Left ventricular systolic function was moderately preserved on average, with a mean preoperative ejection fraction of 51.6%.

**Table 1:** Patients’ characteristics

Variants	(n = 549)
Age (years)	68.6 ± 10.0
Male (n (%))	405 (73.8%)
Body surface area (m^2^)	1.62 ± 0.18
Body mass index (kg/m^2^)	23.4 ± 3.3
Diabetes mellitus (n (%))	281 (51.2%)
Hypertension (n (%))	435 (79.2%)
Dyslipidemia (n (%))	353 (64.3%)
Serum creatinine (mg/dL)	1.54 ± 2.11
Patients undergoing hemodialysis (n (%))	32 (5.8%)
LVEF (%)	51.6 ± 13.4
CCS angina grading scale ≥III	184 (33.5%)
NYHA functional classification ≥III	91 (16.6%)

LVEF: left ventricular ejection fraction; NYHA: New York Heart Association; CCS: Canadian Cardiovascular Society

### Operative parameters

Operative parameters are shown in **Table 2**. In this cohort of 549 patients undergoing multivessel CABG, 16.6% of cases were performed on an urgent or emergent basis. Off-pump CABG was utilized in 60.5% of patients, whereas 14.9% underwent on-pump CABG on the beating heart; the remaining 24.6% underwent on-pump CABG with cardioplegic arrest. Among cases requiring cardiopulmonary bypass, the mean CPB duration was 189 ± 64 min, and the mean aortic cross-clamp (AoX) time (for arrested-heart cases) was 153 ± 56 min. Patients received an average of 3.3 ± 1.0 distal anastomoses per person. In total, 794 distal anastomoses were performed to targets in non–left anterior descending (non-LAD) areas. These comprised 242 anastomoses using in situ ITAs; 27 using free ITAs; 97 using rGEA; 285 using cSVGs; and 143 using NT-SVGs.

**Table 2:** Operative parameters

Variants	(n = 549)
Urgent or emergent (n (%))	91 (16.6%)
CABG procedure	
OPCAB (n (%))	332 (60.5%)
On pump beating CABG (n (%))	82 (14.9%)
On pump arrest CABG (n (%))	135 (24.6%)
CPB time (min)	189 ± 64
AoX time (min)	153 ± 56
Number of distal anastomosis (n)	3.3 ± 1.0
Anastomoses to non-LAD territory (n)	794
Used conduits to non-LAD territory (n)	
In situ ITA	242
Free ITA	27
rGEA	97
cSVG	285
NT-SVG	143

CABG: coronary artery bypass grafting; CPB: cardiopulmonary bypass; cSVG: conventional saphenous vein grafts; ITA: internal thoracic artery; LAD: left anterior descending artery; NT-SVG: no-touch saphenous vein grafts; OPCAB: off-pump coronary artery bypass grafting; rGEA: right gastroepiploic artery; AoX: aortic cross-clamp

### Early clinical outcomes

The early clinical outcomes are shown in **Table 3**. In this cohort, early postoperative outcomes were generally favorable. In-hospital mortality was 1.5% (8 patients). Deep sternal wound infection (mediastinitis) occurred in 4.2% of patients (n = 23), and 10.6% (n = 58) required prolonged mechanical ventilation beyond 24 h. Re-exploration for bleeding was necessary in 1.5% (n = 8), new-onset dialysis due to acute renal failure was required in 0.9% (n = 5), and stroke occurred in 2.6% of patients (n = 14). Among those who received an SVG (397 patients), the incidence of surgical site infection at the vein harvest site was 6.3% (25 cases).

**Table 3:** Early clinic

Variants	(n = 549)	
Hospital mortality (n (%))	8 (1.5%)	
Deep sternum infection (n (%))	23 (4.2%)	
Prolonged ventilation (>24 h) (n (%))	58 (10.6%)	
Reoperation for bleeding (n (%))	8 (1.5%)	
Newly required dialysis (n (%))	5 (0.9%)	
Stroke (n (%))	14 (2.6%)	
SVG harvesting site infection (n (%))	25/397 (6.3%)	
Conventional method	20/281 (7.1%)	p = 0.36 (conventional vs. no-touch)
No-touch method	5/116 (4.3%)	

SVG: saphenous vein grafts

### Late clinical outcomes

**Fig. 2:**
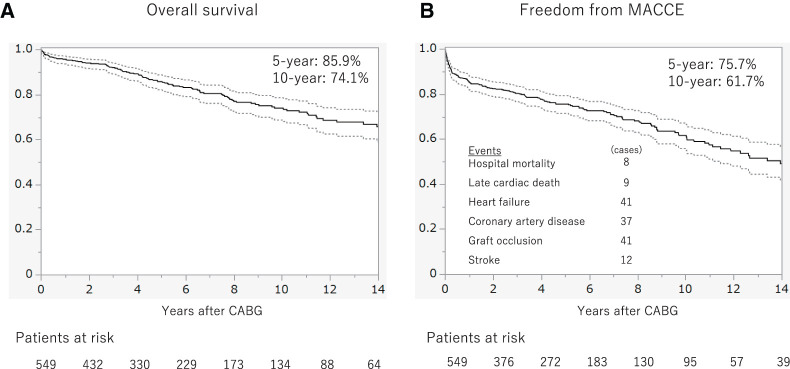
Time-to-event data. Kaplan–Meier survival curves for 549 patients who underwent multivessel CABG between 2002 and 2023. (**A**) Overall survival, with 5- and 10-year survival rates of 85.9% and 74.1%, respectively. (**B**) Freedom from MACCEs, with 5- and 10-year event-free survival rates of 75.7% and 61.7%, respectively. The numbers at risk are shown below each panel. CABG: coronary artery bypass grafting; MACCE: major adverse cardiac and cerebrovascular events

In this cohort, Kaplan–Meier analysis demonstrated overall 5- and 10-year survival rates of 85.9% and 74.1%, respectively (**Fig. 2A**). Freedom from MACCE—defined as cardiac death, heart failure hospitalization, repeat revascularization, graft occlusion, or stroke—was similarly high (75.7% at 5 years and 61.7% at 10 years) (**Fig. 2B**). The mean follow-up duration was 6.5 ± 5.4 years. Details of MACCE included cardiac deaths (n = 34), aortic events (n = 6), and stroke (n = 9).

### Intraoperative graft flow and graft patency

Among the 5 conduit groups (in situ ITA, free ITA, cSVG, NT-SVG, and rGEA), the cSVG exhibited significantly higher mGF compared with in situ ITA, NT-SVG, or rGEA, whereas PI did not differ (**Table 4**).

**Table 4:** Intraoperative graft flow

	To non-LAD area					To LAD area
	In situ ITA	Free ITA	cSVG	NT-SVG	rGEA	In situ ITA-LAD
Mean graft flow (mL/min/anastomosis)	37.3 ± 21.4	49.4 ± 17.0	58.6 ± 38.4*	39.5 ± 20.7	33.0 ± 15.8	50.0 ± 28.4
Pulsatility index	3.6 ± 9.1	2.7 ± 2.3	3.1 ± 1.7	2.5 ± 1.5	2.9 ± 1.1	2.6 ± 1.2

*p <0.01 cSVG vs. in situ ITA, free ITA, NT-SVG, and rGEA.

cSVG: conventional saphenous vein grafts; ITA: internal thoracic artery; LAD: left anterior descending artery; NT-SVG: no-touch saphenous vein grafts; rGEA: right gastroepiploic artery

**Fig. 3:**
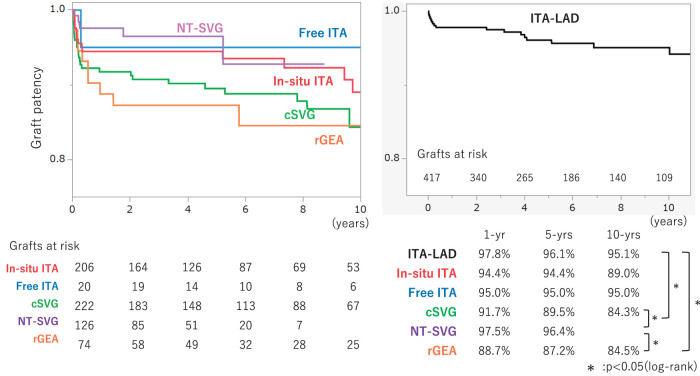
Curves for graft patency. Kaplan–Meier curves comparing graft patency rates by graft type. Curves are shown for ITA (red), free ITA (blue), cSVG (green), NT-SVG (purple), and rGEA (orange). The accompanying table provides the 1-, 5-, and 10-year patency rates for each graft type. Statistically significant differences (p <0.05) between graft types are indicated by an asterisk (*). The patency rates of ITA-LAD, in situ ITA, free ITA, and NT-SVG were comparable. The patency rates of cSVG and rGEA were significantly inferior to those of NT-SVG and ITA-LAD (log-rank p <0.05). ITA: internal thoracic artery; NT-SVG: no-touch saphenous vein graft; cSVG: conventional saphenous vein graft; rGEA: right gastroepiploic artery

In this cohort, all conduit types demonstrated excellent early patency, exceeding ~90% at one year. However, by mid- and long-term follow-up, the Kaplan–Meier curves (**Fig. 3**) differed in graft durability among the 5 conduit types. At 5 years, patency remained highest for arterial and no-touch vein grafts: the NT-SVG achieved a 5-year patency of 96.4%, which was significantly higher than that of cSVG (89.5%) and rGEA (87.2%; p <0.05). NT-SVG exhibited a mid-term patency that was statistically indistinguishable from that of the ITA grafts–both in situ (94.4%) and free (95.0%). By 10 years, ITAs continued to show the highest patency (in situ ITA ~89%, free ITA ~95%). In contrast, cSVG and rGEA grafts exhibited a more pronounced decline over time, with 10-year patency decreasing to approximately 84% in this series. These findings underscore the superior graft longevity associated with the no-touch vein harvesting technique; NT-SVG achieved significantly better patency than CVG and rGEA.

### Comparison of graft patency between cSVG and NT-SVG using propensity score matching

**Fig. 4:**
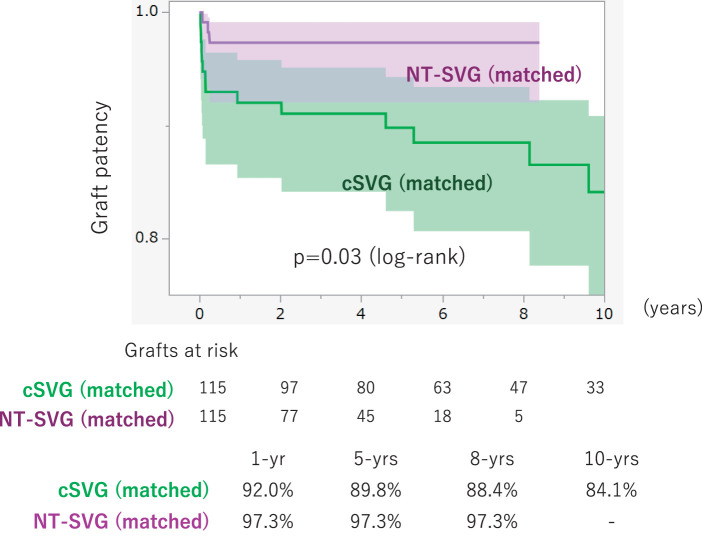
Curves of the graft patency between propensity score-matched cohorts. Kaplan–Meier analysis of graft patency in propensity score–matched cohorts comparing NT-SVG (purple) with cSVGs (green). Shaded areas represent 95% confidence intervals. The NT-SVG group demonstrates significantly higher long-term patency compared with the cSVG group (log-rank p = 0.03). Numbers at risk are shown below the graph. Cumulative graft patency rates at 1, 5, 8, and 10 years are summarized. After matching, the patency of NT-SVG remained superior to that of cSVG. NT-SVG: no-touch saphenous vein graft; cSVG: conventional saphenous vein graft

As shown in **Table 5**, before propensity score matching, the cSVG (n = 285) and NT-SVG (n = 143) groups differed significantly in several baseline characteristics. The NT-SVG cohort had a higher mean body surface area (1.64 ± 0.18 vs. 1.59 ± 0.19 m^2^, p = 0.02) and a greater prevalence of hypertension (87.4% vs. 76.1%, p = 0.0004), dyslipidemia (81.2% vs. 55.0%, p <0.0001), and hemodialysis dependence (11.1% vs. 3.8%, p = 0.0045) compared to the cSVG group, whereas serum creatinine levels were similar between groups (1.87 ± 2.44 vs. 1.58 ± 2.26 mg/dL, p = 0.22). After matching (n = 115 per group), baseline characteristics were well balanced between the two cohorts, except for hypertension (NT-SVG 89.5% vs. cSVG 79.1%, p = 0.03) and the need for hemodialysis (10.4% vs. 2.6%, p = 0.01), which remained more common in the NT-SVG group. The distribution of target vessel stenosis severity did not differ significantly between the two groups, either before or after matching. **Figure 4** shows the graft patency outcomes in the matched population: Kaplan–Meier analysis demonstrated significantly superior long-term patency in the NT-SVG group compared with the cSVG group (log-rank p = 0.03). The NT-SVG cohort maintained a graft patency rate of 97.3% at 1, 5, and 8 years postoperatively, whereas those in the cSVG cohort showed rates of 92.0%, 89.8%, and 88.4% at the respective time points. By 10 years, patency in the cSVG group had declined to 84.1%, while no 10-year data were available for the NT-SVG group (follow-up in the NT-SVG cohort was limited to 8 years).

**Table 5:** Baseline characteristics before and after propensity score matching

Variables	Before matching			After matching		
	cSVG (n = 285)	NT-SVG (n = 143)	p-Value	cSVG (n = 115)	NT-SVG (n = 115)	p-Value
Age (years)	692 ± 9.6	70.3 ± 9.3	0.26	68.7 ± 9.6	69.7 ± 9.7	0.17
Male (n (%))	194 (68.0)	109 (76.2)	0.07	86 (74.7)	88 (76.5)	0.75
Body surface area (m^2^)	1.59 ± 0.19	1.64 ± 0.18	**0.02**	1.62 ± 0.17	1.64 ± 0.18	0.42
Body mass index (kg/m^2^)	23.5 ± 3.5	23.2 ± 3.7	0.41	24.0 ± 2.8	23.2 ± 3.9	0.08
Diabetes mellitus (n (%))	145 (50.8)	84 (58.7)	0.12	55 (47.8)	64 (55.6)	0.23
Hypertension (n (%))	217 (76.1)	125 (87.4)	**0.004**	91 (79.1)	103 (89.5)	**0.03**
Dyslipidemia (n (%))	157 (55.0)	116 (81.2)	**<0.0001**	88 (76.5)	93 (80.8)	0.42
Serum creatinine (mg/dL)	1.58 ± 2.26	1.87 ± 2.44	0.22	1.48 ± 2.31	1.66 ± 2.17	0.54
Hemodialysis (n (%))	11 (3.8)	16 (11.1)	**0.0045**	3 (2.61)	12 (10.43)	**0.01**
Stenosis severity of the target coronary artery (n)						
50% stenosis	1	1		0	1	
75% stenosis	40	27		23	21	
90% stenosis	77	58	0.85	49	51	0.88
99% stenosis	19	20		12	14	
CTO	45	37		31	28	
Unknown	103					

Bold indicates statistical significance (p <0.05).

cSVG: conventional saphenous vein grafts; CTO: chronic total occlusion; NT-SVG: no-touch saphenous vein grafts

## Discussion

Our findings revealed that NT-SVGs achieved superior mid-term patency compared to cSVG and rGEA, while matching the performance of ITA grafts. Kaplan–Meier analysis demonstrated a 5-year graft patency of approximately 96% for NT-SVG, which was significantly higher than that for cSVG (~89%) and rGEA (~87%, p <0.05). Notably, the 5-year patency of NT-SVGs was statistically indistinguishable from in situ and free ITA grafts (≈94%–95%), and this parity appeared to persist to 10 years post-CABG, supporting our hypothesis that NT-SVGs can deliver arterial-like durability. These findings underscore the potential of the NT-SVG technique to bridge the gap between cSVG and ITAs in terms of long-term patency.

Even after propensity score matching, the NT-SVG group in our study contained a significantly higher proportion of patients undergoing dialysis and those with hypertension. SVG patency is known to be markedly impaired in patients undergoing dialysis, with patency rates of 52% and 37% at 1 and 2 years postoperatively, respectively, indicating substantially earlier graft occlusion compared with the general population.^15)^ Hypertension, in addition to being a well-established risk factor for coronary artery disease, has been implicated in reduced SVG patency following CABG.^16)^ Elevated blood pressure may accelerate atherosclerosis and intimal hyperplasia development in grafts, and patients whose postoperative blood pressure remained above 130/80 mmHg demonstrated significantly higher rates of graft occlusion.^16)^ Conversely, adequate blood pressure control has been suggested to attenuate the adverse effects of hypertension on graft outcomes.^17)^ In this study, although the degree of postoperative blood pressure control in hypertensive patients could not be ascertained, the NT-SVG group—despite including a larger proportion of patients with dialysis and hypertension—still exhibited favorable graft patency. Furthermore, stratified analyses according to the presence of hypertension and dialysis revealed no significant differences in graft patency (**Supplementry Fig. 1**). Taken together, these findings suggest that the observed imbalance in patient background after propensity score matching is unlikely to have had a substantial impact on the overall results of this study.

Our results reinforce and extend the observations of previous investigators. Samano et al. famously introduced the NT harvesting technique, hypothesizing that minimal handling and preserved pedicle tissue would improve vein graft outcomes.^7,9,10)^ Randomized controlled trials (RCTs) by Samano et al. have demonstrated that NT-harvested veins have significantly higher patency than conventionally harvested veins in the short term (95.4% vs. 88.9% at 1.5 years) and long term.^18)^ Remarkably, NT-SVG patency rates up to 16 years post-CABG approximate those of the left ITA, the gold-standard graft, in the same patient cohort.^7)^ Subsequent RCTs from other institutions have also reported the efficacy of NT-SVG.^19)^

The improved performance of NT-SVGs can be attributed to multiple mechanisms demonstrated in previous studies. Histological and ultrastructural analyses have shown that NT vein harvesting significantly preserves the endothelial integrity and adventitial architecture of the graft than conventional techniques.^20)^ In conventional vein grafts, aggressive dissection and high-pressure distension cause “dramatic regions of endothelial denudation,” with damage to the intima and vasa vasorum. This injury may promote early graft thrombosis, spasm, and later atherosclerotic changes.^21)^ In contrast, NT-SVGs are harvested with a pedicle of surrounding tissue and without distension, leaving the endothelium essentially intact. Samano et al. noted that preserving the perivascular fat and vasa vasorum of the vein “abolishes venospasm” intraoperatively and helps maintain graft function and flow.^21)^ The pedicled fat mantle releases vasoactive factors (such as adipocyte-derived relaxing factors)^22)^ that promote vasodilation and inhibit intimal hyperplasia, while avoiding overdistension prevents endothelial stretch injury. These mechanistic insights explain the superior long-term patency observed with NT-SVGs and are corroborated by our clinical data. Our finding that NT-SVG patency was comparable to ITA grafts at 5–10 years aligns with the notion that preserving vein graft integrity can dramatically improve its longevity.

It is also important to consider NT-SVGs within the spectrum of second conduit options in CABG. Each graft choice has well-documented advantages and drawbacks. Using a second arterial graft, such as the bilateral ITA (BIMA), has been shown to improve 10-year survival compared to a single ITA (84% vs. 79%).^23,24)^ However, BIMA harvest comes at the cost of increased surgical complexity and a higher risk of sternal wound complications (mediastinitis) due to devascularization of the sternum.^11,25)^ Radial artery (RA) grafts are more resistant to atherosclerosis than saphenous veins and exhibit superior intermediate-term patency. However, the RA is prone to vasospasm and is limited in length.^26)^ The rGEA can be used in situ to revascularize the right coronary territory and has shown acceptable patency (~80%–90% at 5 years and ~60%–70% at 10 years) in some reports. However, its harvest is technically demanding and can significantly prolong operative time.^27)^ In contrast, the saphenous vein is readily available in almost all patients and in generous length; however, conventional harvesting techniques contribute to a well-known decline in vein graft patency to ~40%–60% by 10 years.^6,7)^ Against this backdrop, our study highlights NT-SVG as a conduit that mitigates many of the traditional shortcomings of saphenous veins. The NT technique preserves the natural structure of the vein, thereby narrowing the patency gap between SVGs and ITAs without incurring the donor-site limitations of the RA or the infectious risks associated with BIMA. Our 5-year results showed NT-SVG patency (~96%) equivalent to that of in situ/free ITAs and higher than that of the rGEA, which is a notable achievement given the historic inferiority of vein grafts. These data concur with the pioneering work of Samano et al., who reported that NT vein outcomes can “match” ITA results in the long term.^7)^

A recent European RCT found that the NT vein harvesting technique yielded graft patency rates comparable to those of the conventional technique but was associated with a higher rate of wound complications.^28)^ Given these findings, the authors advised caution when adopting the NT technique. However, despite the valuable insights offered by this study, several methodological limitations must be considered. Notably, the trial had a smaller sample size than earlier RCTs^19)^; each participating center enrolled approximately 100 patients, with roughly half in the NT group and half in the CV group. These cases were likely handled by multiple surgeons at each site, raising concerns about consistency in surgical technique and surgeon proficiency. This issue is especially pertinent for the NT method, which involves preserving PVAT and thus requires special care to avoid twisting or kinking of the graft. Insufficient surgeon experience with the NT technique may have influenced the clinical outcomes observed. Furthermore, because the trial was conducted during the coronavirus disease pandemic, approximately 20% of patients did not complete follow-up, potentially introducing bias into the analysis of long-term graft patency. The investigators also acknowledged that the incidence of vein graft occlusion or stenosis was lower than anticipated. This lower event rate may have reduced the trial’s statistical power, limiting its ability to detect significant differences between the groups. Considering these limitations, the trial’s conclusions regarding its primary endpoints should be interpreted with caution. In the future, additional well-designed, high-powered RCTs with extended follow-up will be essential to provide definitive evidence.

In this study, the proportion of patients with diabetes was higher (51% vs. 27% in Thelin et al.), whereas the cohort reported by Thelin et al. included a higher number of patients with obesity.^28)^ Such differences in baseline characteristics may have influenced the incidence of wound complications. Obesity is a well-known risk factor for infections at the lower limb vein harvesting site, which may explain why Thelin et al. observed a significantly higher rate of wound complications in the NT-SVG group. In contrast, although our study included a higher proportion of patients with diabetes, strict perioperative glycemic control was routinely implemented at our institution. This may explain the comparable incidence of wound complications between the NT-SVG and conventional groups in our study. Furthermore, the mean number of distal anastomoses per case in our study was 3.3 (compared with 2.0 in Thelin et al.), reflecting the treatment of more severe 3-vessel disease. Generally, patients with complex 3-vessel disease requiring multiple distal anastomoses present unfavorable vessel conditions, which are typically associated with reduced vein graft patency. In our study, the preservation of graft quality through the NT harvesting technique may have contributed to favorable patency rates, even under these challenging conditions. In contrast, the randomized trial by Thelin et al. included fewer anastomoses and a more limited disease extent, likely representing more favorable surgical conditions in which conventional vein grafts could achieve good long-term outcomes. Additionally, our study employed rigorous wound management, including the use of closed suction drains and negative pressure wound therapy at the lower limb harvesting site. Placing drains at NT harvesting sites has been shown to eliminate the difference in wound complication rates between NT and conventional techniques.^29)^ Similarly, we believe that our proactive wound management contributed to the acceptable incidence of complications observed in this study. In contrast, Thelin et al. did not specify postoperative wound management strategies, and under standard care conditions, the extensive tissue dissection and residual adipose tissue associated with NT harvesting may have directly translated into a higher incidence of wound complications in the NT-SVG group. Taken together, these differences in patient background, surgical techniques, and postoperative management may explain the discrepancies in outcomes between our study and the report by Thelin et al.

This study has some limitations that should be acknowledged when interpreting the results. First, this was a retrospective, single-center analysis, which inherently carries the risk of selection bias and limits generalizability. The choice of conduit (NT-SVG vs. cSVG vs. other grafts) was not randomized; therefore, there may be underlying differences in patient or disease characteristics influencing graft selection and outcomes.

Second, angiographic follow-up was not performed routinely for all grafts. This incomplete graft assessment means that our patency analysis might overestimate patency rates (if occult asymptomatic occlusions were present in non-imaged patients) or, conversely, could be biased toward detecting failures (if angiography was performed mainly for symptomatic patients). Therefore, the true patency of each conduit in an unselected population may differ. The indications for coronary angiography in this study were as follows:

1. Coronary angiography (or CT) was performed when patients presented with chest symptoms.
2. Patients underwent annual outpatient evaluations including chest radiography, electrocardiography, and echocardiography, and coronary angiography (or CT) was performed when abnormalities were detected.

The rate of angiographic examinations in this study is shown in **Supplementary Table 1**. Previous reports have demonstrated that asymptomatic graft occlusion after CABG occurs in 25% of the patients at 5 years and 39% at 10 years.^30,31)^ Therefore, with our diagnostic strategy, asymptomatic coronary occlusions not detected by electrocardiography or echocardiography were likely missed, suggesting that graft patency may have been considerably overestimated.

Third, the follow-up duration and sample size, though substantial (mean follow-up ~6 years, 549 patients), may be insufficient to detect very late divergences in graft performance (beyond 10–15 years), especially given that the NT technique was introduced more recently at our institution.

Fourth, we did not extensively evaluate clinical endpoints beyond patency (such as freedom from angina, need for repeat revascularization, or long-term survival differences between conduit choices). Graft patency is a crucial surrogate for success in CABG; however, clinical outcomes ultimately determine patient benefit, which was not the primary focus of our analysis. Future studies should thoroughly investigate how the choice of the second graft affects survival outcomes.

Finally, this study has a historical, retrospective design, comparing the outcomes of the cSVG used before 2016 with those of the NT-SVG used after 2016. Meanwhile, starting around 2015, reports began to suggest that implementing dual antiplatelet therapy (DAPT) after CABG improves graft patency, presumably because early SVG occlusion is primarily caused by postoperative thrombosis and DAPT enhances the antiplatelet effect.^32)^ Our institution introduced DAPT around 2015 (although the precise timing is unclear), which may have influenced the graft patency outcomes.

## Conclusions

In this observational study, NT-SVGs appear promising as second conduits in multivessel CABG, with improved vein graft durability observed. The 5-year patency rates of NT-SVGs were comparable to those of arterial grafts and superior to those of conventional vein grafts and the rGEA, suggesting that this technique may serve as a reliable and versatile graft option. However, given the study’s single-arm design and incomplete data, these findings require further validation in comparative or randomized trials. If confirmed in such trials, improved long-term graft patency with NT-SVG could enhance CABG outcomes by reducing the need for repeat interventions and benefiting patients who are not suitable for multiple arterial grafts.

## Declarations

### Data availability

The data generated in this study can be shared upon reasonable request from the corresponding author.

## Supplementary Materials

Supplementary Fig. 1 Curves for the graft patency stratified by clinical risk factors. (**A**) Kaplan–Meier curves for graft patency according to the presence or absence of hypertension. No significant difference was observed between patients with and without hypertension (log-rank p = 0.57). (**B**) Kaplan–Meier curves for graft patency according to the presence or absence of hemodialysis. No significant difference was observed between patients with and without hemodialysis (log-rank p = 0.90). Numbers at risk are shown below each curve.

Supplementary Table 1 Number of cases and contrast-enhanced imaging studies.
