# Supplementary figures and images for: The Clinical Utility of No-Touch Saphenous Vein Grafting as a Second Conduit in Multivessel Coronary Artery Bypass Surgery
Source: Ann Thorac Cardiovasc Surg. 2025 Nov 14;31(1):25-00151. doi: 10.5761/atcs.oa.25-00151 (PMC12620506; doi:10.5761/atcs.oa.25-00151)

Supplemental Fig. 1

(A)

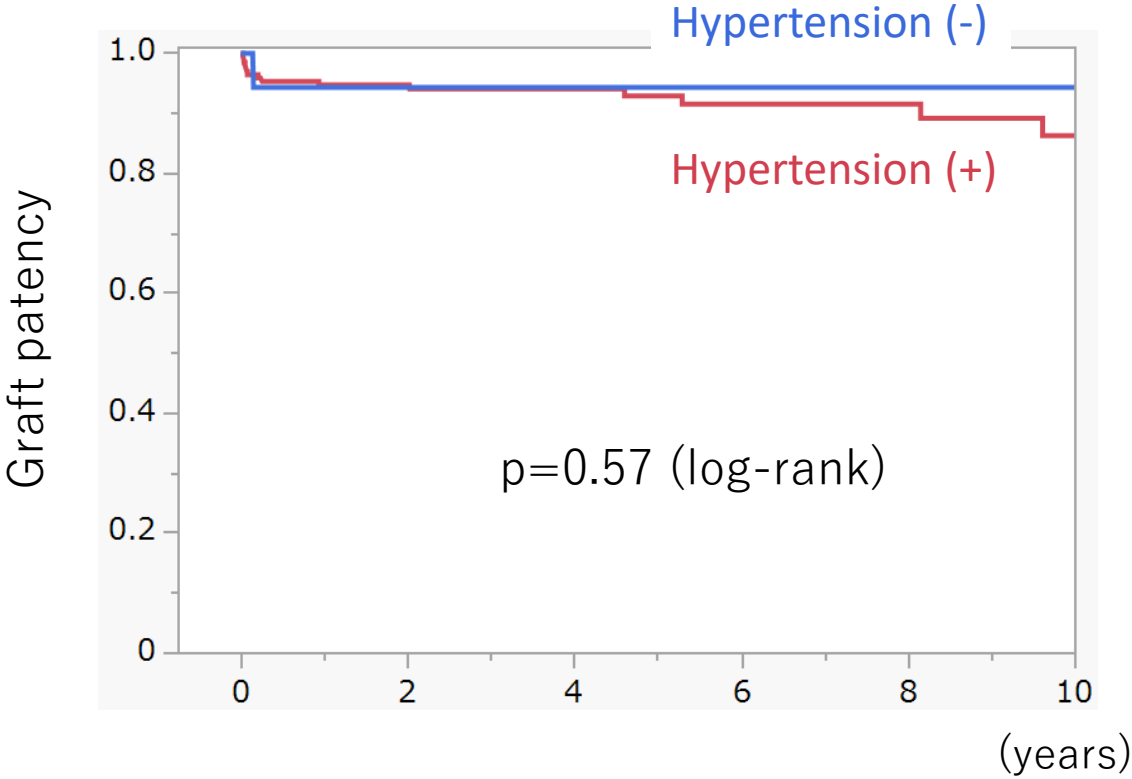

Grafts at risk

|     |     |     |    |    |    |    |
|-----|-----|-----|----|----|----|----|
| (+) | 194 | 142 | 96 | 64 | 42 | 28 |
| (-) | 36  | 32  | 29 | 17 | 9  | 6  |

|     |       |       |        |
|-----|-------|-------|--------|
|     | 1-yr  | 5-yrs | 10-yrs |
| (+) | 94.7% | 92.8% | 86.2%  |
| (-) | 94.2% | 94.2% | 95.2%  |

(B)

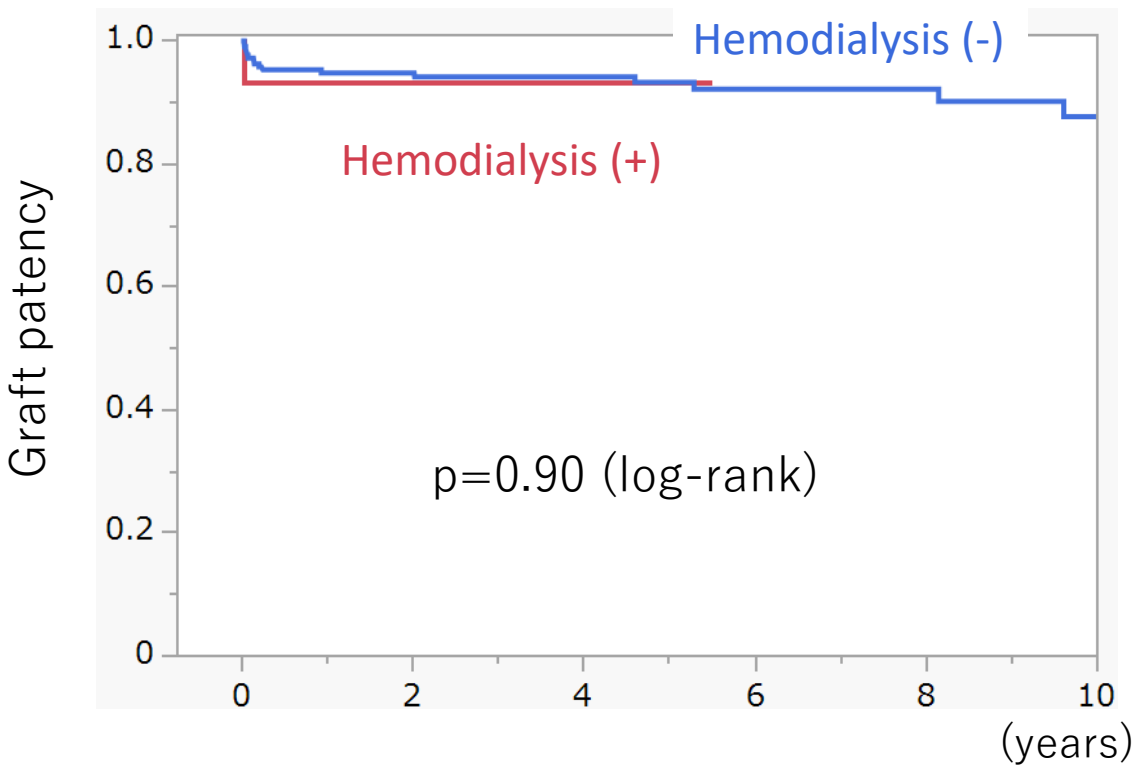

Grafts at risk

|     |     |     |     |    |    |    |
|-----|-----|-----|-----|----|----|----|
| (+) | 15  | 15  | 3   |    |    |    |
| (-) | 215 | 160 | 117 | 80 | 50 | 33 |

|     |       |       |        |
|-----|-------|-------|--------|
|     | 1-yr  | 5-yrs | 10-yrs |
| (+) | 94.7% | 92.8% | -      |
| (-) | 94.7% | 93.2% | 87.6%  |

Supplement: Supplementary Fig. 1 — Curves for the graft patency stratified by clinical risk factors. (A) Kaplan–Meier curves for graft patency according to the presence or absence of hypertension. No significant difference was observed between patients with and without hypertension (log-rank p = 0.57). (B) Kaplan–Meier curves for graft patency according to the presence or absence of hemodialysis. No significant difference was observed between patients with and without hemodialysis (log-rank p = 0.90). Numbers at risk are shown below each curve. [file atcs-31-1-25-00151-s001.pdf]
