# Supplementary material for: The Clinical Utility of No-Touch Saphenous Vein Grafting as a Second Conduit in Multivessel Coronary Artery Bypass Surgery
Source: Ann Thorac Cardiovasc Surg. 2025 Nov 14;31(1):25-00151. doi: 10.5761/atcs.oa.25-00151 (PMC12620506; doi:10.5761/atcs.oa.25-00151)
Supplement: Supplementary Table 1 — Number of cases and contrast-enhanced imaging studies. [file atcs-31-1-25-00151-s002.pdf]

Supplementary Table 1. Number of cases and contrast-enhanced imaging studies

	1 month	5 years	10 years
ITA-LAD	417/549 (75.9%)	51/229 (22.2%)	13/109 (11.9%)
In-situ ITA	206/242 (85.1%)	34/108 (31.4%)	10/53 (18.8%)
Free ITA	20/27 (74.0%)	4/12 (33.3%)	1/6 (16.6%)
cSVG	222/285 (77.8%)	40/131 (30.5%)	11/67 (16.4%)
NT-SVG	126/143 (88.1%)	4/35 (11.4%)	
rGEA	74/97 (76.2%)	12/40 (30%)	4/25 (16.0%)

The denominator corresponds to the number of cases, the numerator to the number of contrast examinations, and percentages are presented in parentheses.

cSVG, conventional saphenous vein grafts; ITA, internal thoracic artery; LAD, left anterior descending artery; NT-SVG, no-touch saphenous vein grafts; rGEA, right gastroepiploic artery.
